# Hydrogen peroxide/ATR-Chk2 activation mediates p53 protein stabilization and anti-cancer activity of cheliensisin A in human cancer cells

**DOI:** 10.18632/oncotarget.1780

**Published:** 2014-02-14

**Authors:** Jingjie Zhang, Guangxun Gao, Liang Chen, Jingxia Li, Xu Deng, Qin-shi Zhao, Chuanshu Huang

**Affiliations:** ^1^ Nelson Institute of Environmental Medicine, New York University School of Medicine, Tuxedo, NY, USA; ^2^ Department of Hematology, Xijing Hospital, Fourth Military Medical University, Xi'an, Shaanxi, China; ^3^ State Key Laboratory of Phytochemistry and Plant Resources in West China,Kunming Institute of Botany, Chinese Academy of Sciences, Kunming, China

**Keywords:** Cheliensisin A, Apoptosis, colon cancer, p53, chemotherapeutic agent

## Abstract

Cheliensisine A (Chel A) as a novel styryl-lactone isolated from Goniothalamus cheliensis Hu has been indicated to be a chemotherapeutic agent in Leukemia HL-60 cells. However, its potential for cancer treatment and the underlying mechanisms are not deeply investigated to the best of our knowledge. Current studies showed that Chel A could trigger p53-mediated apoptosis, accompanied with dramatically inhibition of anchorage-independent growth of human colon cancer HCT116 cells. Further studies found that Chel A treatment resulted in p53 protein stabilization and accumulation via the induction of its phosphorylation at Ser20 and Ser15. Moreover, Chel A-induced p53 protein accumulation and activation required ATR/Chk2 axis, which is distinct from the mechanism that we have most recently identified the Chk1/p53-dependent apoptotic response by Chel A in normal mouse epidermal Cl41 cells. In addition, our results demonstrated that hydrogen peroxide generation induced by Chel A acted as a precursor for all these signaling events and downstream biological effects. Taken together, we have identified the Chel A as a new therapeutic agent, which highlights its potential for cancer therapeutic effect.

## INTRODUCTION

Cancers of the colon and rectum continue to be the third most common fatal cancers in the United States, and approximately account for 10% of the total cancer deaths [1]. In an effort to fight against such diseases, targeted cancer therapies, which normally employing drugs block the growth of cancer by interfering with specific molecules involved in tumor growth and progression, turns out to be an effective strategy [2]. The p53 tumor suppressor pathway is dysfunctional in some colorectal cancers [3], and to this end, identification of therapeutics that is capable of specifically re-activating p53-mediated apoptosis affords a tempting approach to treat colon cancer.

Cheliensisine A (Chel A) as a novel styryl-lactone isolated from *Goniothalamus cheliensis* has been demonstrated to be cytotoxic to human promyelocytic leukemia HL-60 cells[4]. Mechanistic insight revealed that this natural product could trigger apoptosis via downregulation of Bcl-2 expression [5]. Furthermore, a recent study in our laboratory has indicated the inhibitory effect of Chel A on EGF-induced cell transformation in JB6 Cl41 cells via the activation of p53-dependent pathway [6]. Taken together, previous studies have identified Chel A as a dual chemotherapeutic and chemopreventive agent that could be potentially used for cancer treatment and prevention. However, the effect and mechanism of Chel A on colon cancer has not been identified yet. Therefore, colon cancer HCT116 cells were employed to evaluate the potential chemotherapeutic effect of Chel A. Briefly, our results found that Chel A was capable of activating p53-mediated apoptosis in HCT116 cells, which in turn resulted in the inhibition of anchorage-independent growth of HCT116 cells. Further studies suggested that Chel A could stabilize and activate p53 via the phosphorylation at Ser20 and Ser15, and its activation could be via the binary pathways, i.e. ATR/p53 signaling and ATR/Chk2/p53 axis. Finally, it was found that hydrogen peroxide generation induced by Chel A acted as a precursor for all these signaling events and downstream biological effects. Collectively, this study indicated the chemotherapeutic effect and the molecular mechanisms underlying Chel A inhibition of colon cancer anchorage-independent growth.

## RESULTS

### Chel A inhibited cell viability and anchorage-independent growth of colon cancer HCT116 cells

**Fig 1:**
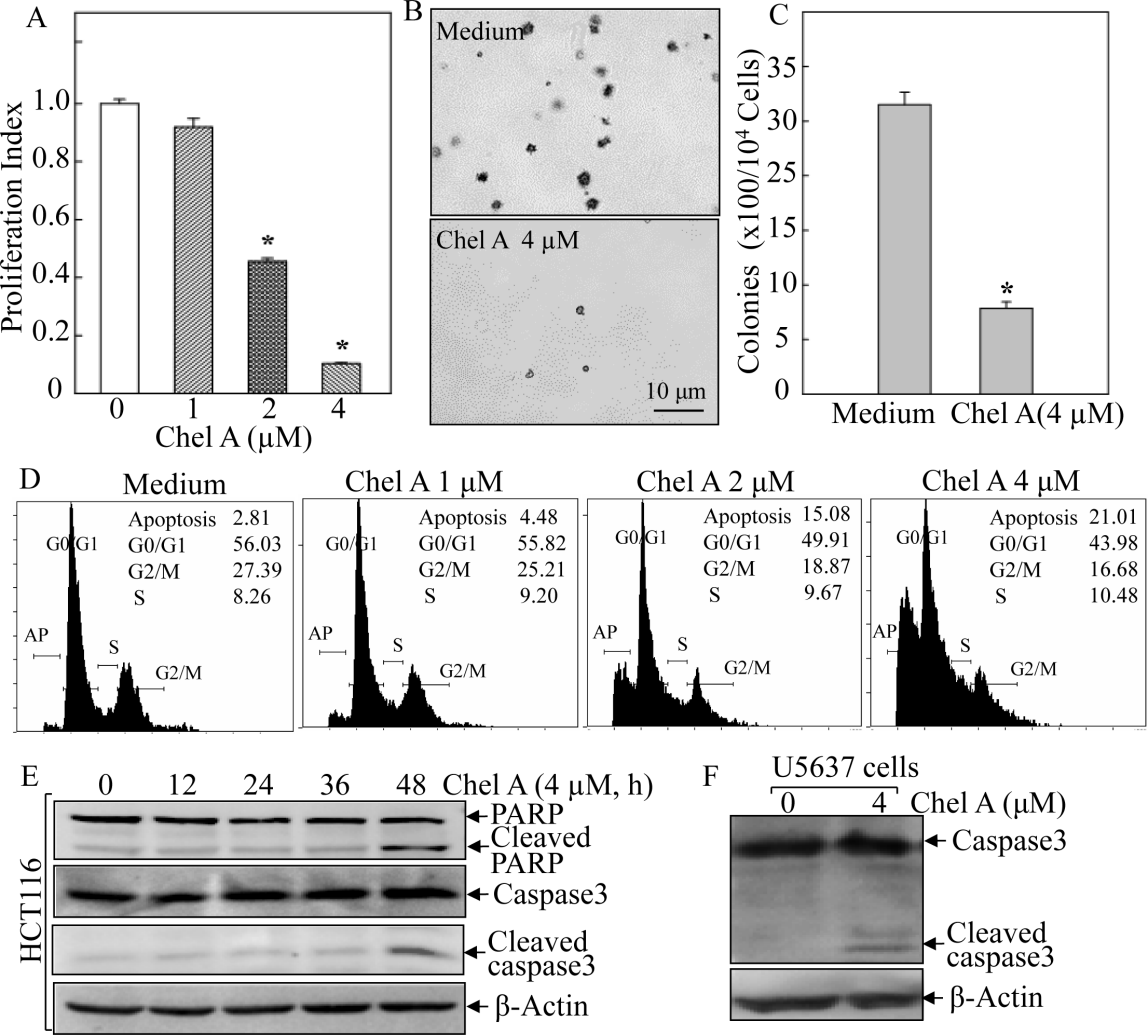
Chel A inhibited cell viability and anchorage-independent growth via induction of apoptosis in human colon cancer HCT116 cells (A) HCT116 cells were treated with Chel A for cell proliferation assay as described in “Material and Methods”. After treated for 48 h, cell proliferation was measured by using Cell Titer-GloLuminescent Cell Viability Assay kit. The results are expressed as relative luminescence signal to medium control (proliferation index). Each bar indicates the mean and SD of triplicate assays. The symbol (*) indicates a significant decrease as compared with that of medium control (*P*<0.05). (B&C) HCT116 cells were exposed to indicate concentrations of Chel A in soft agar as described in Material and Methods. After being cultured in 37°C with 5% CO_2_ for 3 weeks, the colony formation was observed under inverted microscope and photographed (B). The number of colonies was scored, and presented as colonies per 10^4^ seeded cells (C). The symbol (*) indicates a significant decrease as compared with that of vehicle control (*P* <0.05). Each bar indicates the mean and SD from three independent experiments. (D-F) HCT116 cells (D & E) or U5637 cells (F) were cultured in each well of a six-well plate with McCoy's 5A medium containing 10% FBS at 37°C overnight. After synchronization of cells by culturing in McCoy's 5A medium containing 0.1% FBS for 24 hours, the cells were treated with various concentrations Chel A as indicated, for 48 hours (D) or with 4.0 ¼M Chel A for indicated time periods (E) or for 36 hrs (F). The cells as indiacted were collected and subjected to flow cytometry assay (D) and Western blot assay (E & F). The result was representative one from three independent experiments.

Chel A has been shown possessing cytotoxicity in human leukemia HL-60 cells [5]. However, the anti-cancer activity of Chel A on colon cancer has not been explored yet. Therefore, we first assessed the effect of Chel A on cell viability of colon cancer cells using ATPase assay. HCT116 colon cancer cell line was selected and cultured with a range of Chel A doses (1.0-4.0 μM) for 48 hrs. As shown in Fig. 1A, a significant reduction of cell viability was observed in a dose-dependent manner, and IC_50_ of Chel A on HCT116 cells was approximately 2.0 μM. Next, soft agar assay was employed to determine the inhibitory effect of Chel A on anchorage-independent growth (colony formation). The results showed that anchorage-independent growth of HCT116 cells was significantly inhibited following 4 μM Chel A treatment (Figs. 1B and 1C). These results clearly demonstrated the anti-cancer effect of Chel A in human colon cancer cells.

### Chel A treatment exerted its anti-cancer effect via induction of caspase-dependent apoptosis in HCT116 cells

Cancer therapeutic agents could exert their anti-cancer effect via either causing cell growth arrest or inducing cancer cell apoptosis. Thus, we used flow cytometry assay to see if Chel A inhibited cell colony formation via interfering with cell cycle progression in HCT116 cells. The HCT116 cells were treated with Chel A and cell death was examined by flow cytometry. As shown in Fig. 1D, the HCT116 cells were treated with Chel A at concentrations of 0, 1.0, 2.0, and 4.0 μM for 48 hrs. High-resolution flow cytometric analysis of PI-stained nuclei showed that sub-G1 DNA content (cell death peak) at 48 hrs increased substantially compared to the negative control in a dose-dependent manner (Fig. 1D), revealing that Chel A's inhibitory effect on cell colony formation acted through inducing cell apoptosis rather than block cell cycle progression. Considering that poly (ADP-ribose) polymerase (PARP) cleavage and caspase activation often correlate with apoptosis [7], western blot analysis was performed to check the induction of cleavage of caspase-3 and PARP. The data indicated that an increased cleavage of caspase-3 and PARP was observed by Chel A in HCT116 cells in a time-dependent manner (Fig. 1E). The induction of apoptosis by Chel A was also extended to be determined in human bladder cancer U5637 cells (Fig. 1F). These results strongly demonstrated that Chel A treatment could result in the caspase-dependent apoptosis in HCT116 cells.

### p53 was pivotal in Chel A-induced cell apoptosis

**Fig 2:**
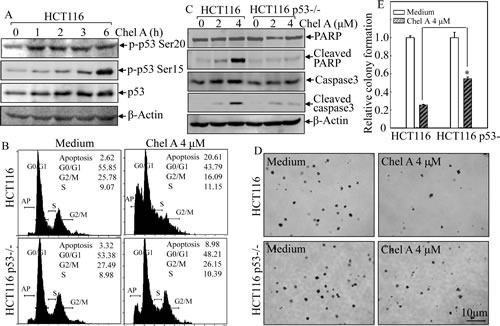
p53 induction mediated Chel A-induced apoptosis (A) HCT116 cells were treated with Chel A for indicated time periods and cell extracts were subjected to Western blotting for the detection of phosphorylated and total p53 protein expression. β-Actin was used as a control for protein loading. (B&C) HCT116 p53+/+ and p53−/− cells were seeded into six-well plates. The cells were treated with Chel A for 48 hrs and then subjected to flow cytometry assay (B) or treated with Chel A for 48 hrs and then subjected to Western blotting to determine the expression and cleavages of PARP and Caspase 3. β-Actin was used as a control for protein loading (C). The result showing was a representative one from three independent experiments. (D&E) HCT116 p53+/+ and p53−/− cells were exposed to indicate concentrations of Chel A in soft agar as described in Material and Methods. After being cultured in 37℃ with 5% CO_2_ for 3 weeks, the colony formation was observed under inverted microscope and photographed (D). The relative colony formation was presented as the colony in Chel A-treated group relative to the vehicle control in HCT116 p53+/+ and p53−/− cells, respectively (E). The symbol (*) indicates a significant increase in HCT116 p53−/− cells with treatment of Chel A as compared with that in HCT116 p53+/+ cells treated with Chel A (p <0.05). Each bar indicates the mean±SD of three independent experiments.

p53, as a tumor suppressor, has been reported to be involved in caspase-dependent apoptosis [8]. Hence, to further explore the mechanism of Chel A's anti-cancer effect, we performed Western blot to check whether p53 participated in the Chel A-induced cell apoptosis. Phosphorylated status of p53 at Ser20 and Ser15, which are positively associated with protein stabilization and apoptotic induction [9, 10], and total p53 protein expression, were examined in Chel A-treated HCT116 cells. The data showed that the phosphorylation of p53 at Ser20 was significantly upregulated following 1 hr and thereafter exposure, whereas the phosphorylation of p53 at Ser15 was almost slightly increased at early (≤3 hrs) time upon treatment and increased significantly at 6 hrs of the treatment. The total p53 expression exhibited a steady increasing trend in response to Chel A treatment, reaching maximum at 2-6 hours (Fig. 2A). Since phosphorylation at Ser20 normally contributes to the stabilization of p53 protein [9], which in turn can induce the phosphorylation at Ser15 [11], we anticipated that Chel A-induced p53 activation in HCT116 cells was likewise based on its phosphorylation at Ser20.

In an effort to further substantiate if p53 activation is necessary for Chel A-induced apoptosis, p53−/− HCT116 cells were employed. As shown in Fig. 2B, the apoptosis induction by Chel A observed from flow cytometric analysis was much lower in HCT116 p53−/− cells compared to that in HCT116 cells with wild-type p53 cells. Further Western blot analysis demonstrated that p53 knockout was capable of blocking Chel A-induced cleavage of PARP and caspase-3, suggesting that p53 activation was critical for apoptotic induction by Chel A in HCT116 cells. Additionally, p53 knockout at least partially attenuate the inhibition of colony formation by Chel A (Figs. 2D & 2E). These results indicated that p53 participated in Chel A's effect on apoptotic induction and further on inhibition of cancer cell anchorage-independent growth.

### Chel A inhibited p53 protein degradation

**Fig 3:**
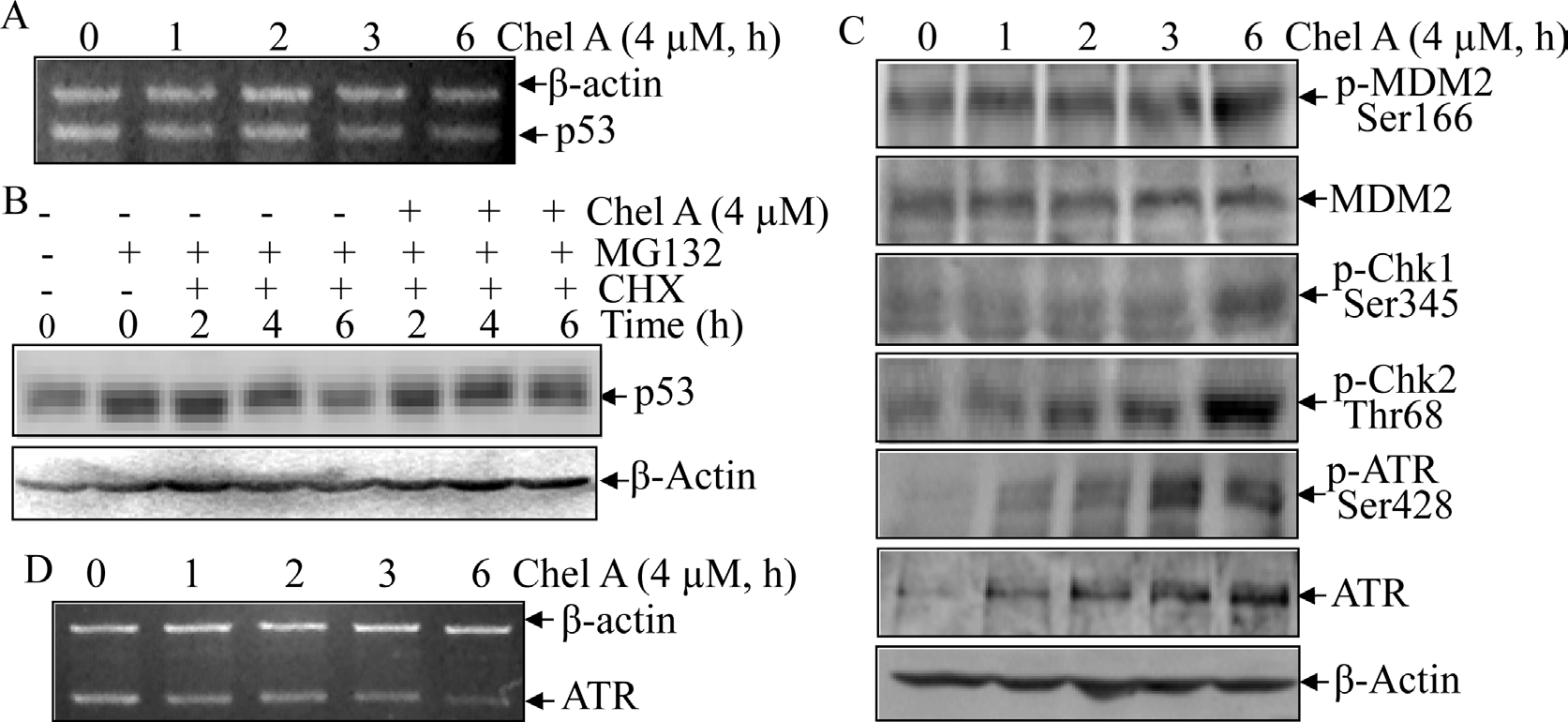
p53 protein induction by Chel A was via the inhibition of p53 protein degradation (A) HCT116 cells were treated with Chel A for indicated time periods, and p53 mRNA was measured by RT-PCR. (B) HCT116 cells were pretreated with MG132 for 4 hrs, followed by exposure with CHX combined with Chel A or CHX alone as indicated. Then cell extracts were subjected to Western Blotting and β-Actin protein expression was used as a protein loading control. (C) HCT116 cells were exposed to Chel A for indicated time periods, and cell extracts were subjected to Western Blotting and β-Actin protein expression was used as a protein loading control. (D) HCT116 cells were treated with Chel A for indicated time periods, and ATR mRNA was evaluated by RT-PCR.

To delineate the molecular mechanism responsible for p53 protein upregulation by Chel A, we first examined the effect of Chel A on p53 mRNA level in HCT116 cells. The results from RT-PCR revealed that Chel A exerted no impact on p53 mRNA level within 6 hrs along the time course (Fig. 3A). Hence, p53 protein induction by Chel A might be regulated at the protein level.

To clarify whether Chel A could prevent p53 protein from degradation, proteasome inhibitor MG132 was employed to pretreat HCT116 cells. MG132 was then removed and the synthetic inhibitor cycloheximide(CHX) was added to the cells alone or in combination with Chel A for various time periods. Such an experimental system could afford insight into effect of Chel A on the dynamic of p53 protein degradation. As shown in Fig. 3B, p53 protein level at 4 hrs and 6 hrs in cells co-treated with Chel A and CHX is much higher than those observed in the cells treated with CHX alone, suggesting that Chel A treatment accumulate p53 via inhibiting p53 protein degradation.

### Chk2 participated in p53 induction during Chel A treatment

Several proteins, such as MDM2, Chk1/2, and ATR are capable of regulating p53 at the protein level [10, 12]. MDM2, as p53-specific E3 ubiquitin ligase, can promote p53 protein degradation [12]. Posttranslational phosphorylation of the MDM2 protein at Ser166 has been reported to contribute to p53 protein degradation [13]. Thus, MDM2 phosphorylation at Ser166 was examined in Chel A-treated HCT116 cells. The data demonstrated that total MDM2 protein expression was not affected by Chel A, whereas phosphorylation at Ser166 was slightly upregulated at 6 hrs following Chel A treatment (Fig. 3C). These results indicated that Chel A exerted no inhibitory effect on MDM2 phosphorylation at Ser166, thereby not accounting for accumulation of p53 protein in Chel A-treated HCT116 cells.

In addition to MDM2, Chk1 and Chk2 are likewise the potential mediators that stabilize and activate p53 via inducing phosphorylation of p53 protein [14]. Given that the phosphorylation of Chk1 at Ser345 or of Chk2 at Thr68 is required for their activation [15, 16], we thus examined the phosphorylated status at these two sites of Chk1 and Chk2, respectively, upon Chel A treatment. Western blot analysis showed that phosphorylation of Chk2 at Thr68 was induced as early as 2 hrs and reaching the peak at 6 hrs following Chel A treatment, whereas an elevated phosphorylation of Chk1 at Ser345 was only slightly increased at 6 hrs of Chel A treatment (Fig. 3C). Due to the fact that phosphorylation of p53 was induced at an early stage (approximately 2 hrs), Chk1 was excluded from directly participation in p53 induction, whereas Chk2 might play a role in p53 stabilization and transactivation.

### Chk2 knockout attenuated p53 activation, cell apoptosis, and the inhibition of colony formation by Chel A in HCT116 cells

**Fig 4:**
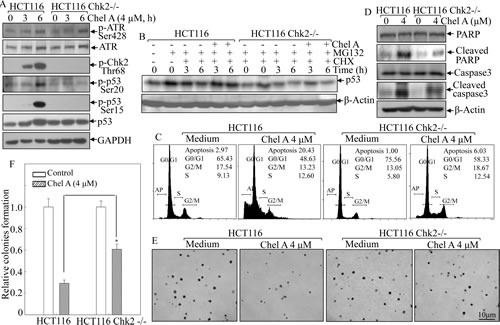
Chk2 mediated the biological effect of Chel A in HCT116 cells (A) HCT116 Chk2−/− cells and its parental wild type cells were treated with Chel A as indicated, and the cell extracts were subjected to Western Blotting for determination of the protein expressions using specific antibodies. β-Actin was used as protein loading controls. (B) HCT116 Chk2−/− and its parental wild type cells were pretreated with MG132 for 4 hrs, followed by exposure with CHX combined with Chel A or CHX alone as indicated. Then cell extracts were subjected to Western Blotting and β-Actin protein expression was used as a protein loading control. (C&D) HCT116 Chk2+/+ and Chk2−/− cells were treated with Chel A (4.0 ¼M) for 48 hrs. Cells were then collected for flow cytometry assay (C) or subjected to Western blotting as indicated (D). The result showing was a representative one from three independent experiments. (E&F) HCT116 Chk2+/+ and Chk2−/− cells were exposed to indicate concentrations of Chel A in soft agar. After being cultured in 37°C with 5% CO_2_ for 3 weeks, the colony formation was observed under inverted microscope and photographed (E). The relative colony formation was presented as the colony in Chel A-treated group relative to the vehicle control in HCT116 Chk2+/+ and Chk2−/− cells, respectively (F). The symbol (*) indicates a significant increase in HCT116 Chk2−/− cells with Chel A treatment as compared with that in HCT116 Chk2+/+ cells treated with Chel A (*P*<0.05). Each bar indicates the mean±SD of three independent experiments

To substantiate the role of Chk2 in p53 activation, we used HCT116 Chk2−/− cells to analyze protein levels of p53 protein phosphorylation and expression following Chel A treatment. As shown in Fig. 4A, phosphorylated Chk2 protein was depleted in HCT116 Chk2−/− cells compared to that in Chk2+/+ cells. Importantly, Chel A-induced p53 protein expression was blocked in Chk2−/− cells, and the phosphorylation of p53 at Ser20 and Ser15 were almost abrogated by Chk2 knockout. Since phosphorylation at ser20 normally contributes to the stabilization of p53 protein [8], which in turn can induce the phosphorylation at ser15 [10], we anticipated that Chk2 mediated Chel A-induced p53 stabilization and protein accumulation by regulating p53 phosphorylation at Ser15 and Ser20. In an effort to further elucidate whether Chk2-mediated p53 accumulation was resulted from the prevention of p53 protein degradation, we utilized HCT116 Chk2−/− cells and aforementioned proteasome inhibitor MG132 and protein synthesis inhibitor CHX. The results showed that Chel A-inhibition of p53 protein degradation in HCT116 Chk2+/+ cells was not observable in HCT116 Chk2−/− cells under same experimental conditions (Fig. 4B), further revealing that Chk2 mediated Chel A-induced p53 protein expression via increasing its stability. Consistently, Chk2-deletion abrogated apoptosis (Fig. 4C), cleavages of caspase-3 and PARP (Fig. 4D), as well as inhibition of anchorage-independent growth (Figs. 4E&4F) in HCT116 cells. These results strongly indicated that Chk2 expression was crucial for p53 phosphorylation at Ser 20 and Ser15, which in turn increased p53 protein stabilization, and further led to apoptosis following Chel A treatment.

### ATR deletion crippled Chk2-mediated p53 protein phosphorylation, apoptosis, and inhibition of anchorage-independent growth by Chel A in HCT116 cells

In addition to Chk1 and Chk2, ATR is a P-I3 kinase-related kinase (PIKK) family member capable of activating p53 directly or indirectly via inducing posttranslational phosphorylation of p53 protein at Ser15 in response to some stimuli [16]. Previously report also demonstrates that ATR activation is associated with its phosporylation at Ser428 [16]. Thus, in an effort to evaluate the role of ATR in p53 expression, phosphorylated ATR at Ser428 was examined in Chel A-treated HCT116 cells. Western blot analysis revealed that ATR phosphorylation at Ser428 was induced as early as 1 hr and reached maximal at 3 hrs following Chel A treatment with gradually increased ATR protein expression (Fig. 3C). The results from RT-PCR indicated that ATR mRNA was not induced by Chel A treatment (Fig. 3D), suggesting that Chel A-induced ATR protein expression was regulated at either protein translation and/or protein degradation, rather than protein transcription and mRNA stability. Collectively, our results demonstrated that ATR was activated by Chel A, and further revealing potential contribution of ATR to p53 protein induction and function.

**Fig 5:**
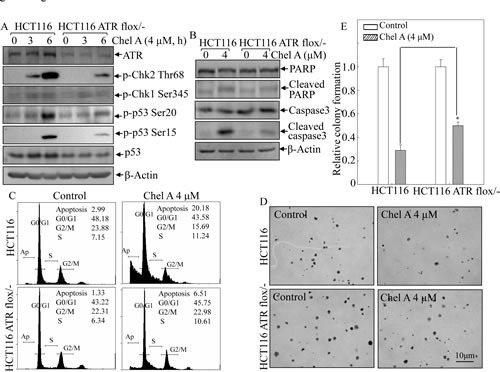
ATR conditional knockout attenuated the biological effect of Chel A in HCT116 cells (A) HCT116 ATR flox^/−^ cells and its parental wild type cells were treated with Chel A as indicated, and the cell extracts were subjected to Western blotting for determination of the protein expressions using specific antibosies. β-Actin was used as protein loading controls. (B&C) HCT116 ATR flox^/-^ and wild type cells were were treated with Chel A for 48 hrs. The cells were then collected and subjected to flow cytometry analysis (B), or subjected to Western blotting(C). The result showing was a representative one from three independent experiments. (D&E), HCT116 ATR flox/- and its parental wild type cells were exposed to indicated concentrations of Chel A in soft agar. After being cultured in 37°C with 5% CO_2_ for 3 weeks, the colony formation was observed under inverted microscope and photographed (D). The relative colony formation was presented as the colony in Chel A-treated group relative to the vehicle control in HCT116 ATR^+/+^ and ATR flox^/-^ cells, respectively (E). The symbol (*) indicates a significant increase in HCT116 ATR flox^/-^ cells with Chel A treatment as compared with that in HCT116 cells treated with Chel A (*P*<0.05). Each bar indicates the mean±SD of three independent experiments.

To understand the role of ATR in p53 protein induction by Chel A, HCT116 cells with conditional knockout of ATR were used. As shown in Fig. 5A, ATR was verified to be conditional depleted in HCT116 cells. Notably, Chel A-induced phosphorylation of Chk2 at Thr68 and of p53 at Ser20 and Ser15, as well as p53 protein induction were impaired in HCT116 ATR flox/- cells in comparison to these in parental HCT116 cells. In contrast, the deletion of Chk2 did not show observable reduction of either ATR phosphorylattion at Ser428 or ATR protein expression following Chel A treatment (Fig. 4A). These data strongly suggested that ATR served as an upstream factor regulating Chk2 activation and played a key role in mediating Chel A-induced Chk2 activation and p53 protein stabilization. Moreover, ATR conditional knockout also abolished apoptosis (Fig. 5C), cleavages of caspase-3 and PARP (Fig. 5B) induced by Chel A treatment, as well as Chel A inhibition of anchorage-independent growth (Figs. 5D&5E) in HCT116 cells. Collectively, our results indicated that upon Chel A treatment, ATR activated Chk2, and in turn mediated p53 phsphorylation and stabilization, subsequently resulting cancer cell apoptosis and inhibition of anchorage-independent growth of cancer cells.

### Hydrogen peroxide generation by Chel A initiated ATR/Chk2 activation, p53 protein induction, apoptosis and inhibition of anchorage-independent growth

**Fig 6:**
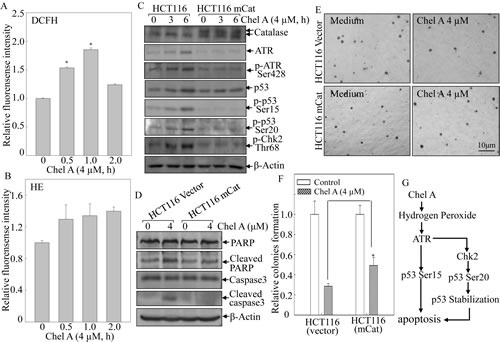
mCat overexpression blocked the biological effect of Chel A-induced hydrogen peroxide in HCT116 cells (A&B) HCT116 cells were pretreated with 2',7'-dichlorfluorescein-diacetate (DCFH) (A) ordihydroethidium (HE) (B), respectively, for 0.5 hr, followed by being treated with Chel A for indicated time periods. Then cells were washed with PBS, and subjected to fluorescence intensity detection. The results were shown as the induction of each time point relative to 0 h(Relative fluorescence intensity). The symbol (*) indicates a significant increase as compared with that at 0 h (P <0.05). Each bar indicates the mean±SD from three independent experiments. (C&D) HCT116 mCat and its parental HCT116 (vector) cells were treated with Chel A for 0-6 hrs, and the cell extracts were subjected to Western blotting. β-Actin was used as protein loading controls. (E&F) HCT116 mCat and HCT116 (vector) cells were treated with indicated concentrations of Chel A in soft agar. After being cultured in 37°C with 5% CO_2_ for 3 weeks, the colony formation was observed under inverted microscope and photographed (E). The relative colony formation was presented as the colony in Chel A-treated group compared with vehicle control in HCT116 mCat and HCT116 (vector) cells, respectively (F). The symbol (*) indicates a significant increase in HCT116 mCat cells treated with Chel A as compared with that of HCT116 (vector) cells treated with Chel A (p <0.05). Each bar indicates the mean±SD of three independent experiments. (G)The diagram indicates mechanisms responsible for Chel A-induced apoptosis in HCT116 cells.

Reactive oxygen species (ROS) has been extensively documented to induce DNA damage, which can activate kinases including ATR and Chk2 [17, 18]. Hence, 2',7'-dichlorofluorescein diacetate (DCFH-DA) and hydroethidine (HE) were employed to determine the generation of intracellular hydrogen peroxide and superoxide anion following HCT116 treated with Chel A. As illustrated in Figs. 6A& 6B, the generation of hydrogen peroxide was significantly increased and reached maximual at 1 hr following Chel A treatment, whereas the increasing tendency of superoxide level was not pronounced under the same experimental conditions. To further assess whether generation of hydrogen peroxide promote the ATR/Chk2 activation and p53 protein induction in response to Chel A stimulation, HCT116 cells were stably transfected with mitochondria catalase (mCat) and ectopic expression of mCat in HCT116 mCat cells was identified as shown in Fig. 6C. mCat overexpression attenuated the phosphorylation of ATR at Ser428, Chk2 phosphorylation at Thr68, and p53 phosphorylation at Ser20 and Ser15, as well as p53 protein induction in comparison to these in parental HCT116 (vector) cells, suggesting that elevated intracellular level of hydrogen peroxide was required for the activation of ATR/Chk2, and p53 protein induction by Chel A. Importantly, the inhibitory effect of Chel A on anchorage-independent growth of HCT116 cells was also blocked by mCat ectopic expression. These results demonstrated that hydrogen peroxide generation by Chel A treatment initiated phosphorylation and activation of ATR/Chk2 cascade, further leading to p53 phosphorylation and protein accumulation, and subsequently resulting in apoptosis and inhibition of anchorage-independent growth of cancer cells.

## DISCUSSION

The styryl-lactone Chel A has been shown to trigger apoptosis in human promyelocytic leukemia HL-60 cells by downregulating Bcl-2 expression, thereby exhibiting the potential as a chemotherapeutic agent in treating human leukemia cells [4, 5]. Our most recently study also demonstrates the chemopreventive effect of Chel A by investigating its inhibition of EGF-induced mouse epidermal Cl41 cell transformation with apoptotic induction [6]. However, the therapy potential and its mechanism of Chel A in colon cancer have not been identified yet. In the present study, we have unveiled another biological function of Chel A that inducing apoptosis and inhibition of cell colony formation in HCT116 cell lines. Further mechanistic study manifested that Chel A-elicited apoptosis was via hydrogen peroxide/ATR/Chk2/p53 pathway, which is distinctive from the mechanisms responsible for Chel A cancer chemotherapeutic and chemopreventive effect in HL-60 cells and JB6 Cl41 cells, respectively. This finding indicated that Chel A could also potentially serve as intervention for colon cancer therapy.

The p53 tumor suppressor pathway is dysfunctional in most colorectal cancers [3], and thus employing rational attempts reversing p53 downregulation in cancer cells by impairing p53 degradation would be a tempting strategy for colon cancer therapy. Intriguingly, we found that Chel A-induced apoptosis in HCT116 cells was mediated by accumulation of p53 protein expression. Particularly, Chel A treatment could promote the stabilization and accumulation of p53 protein by inducing its phosphorylation at Ser20, which in turn contributes to p53 protein induction by increasing its phosphorylation at Ser15. Moreover, our results strongly indicate that Chel A treatment regulates p53 by preventing p53 proteins from degradation.

Regulation of p53 protein degradation may result from several factors, typically including MDM2, Chk1/2, and ATR [10, 12]. Amongst these factors, the results from our studies clearly indicated that MDM2 and Chk1 were denied as direct regulators involved in p53 upregulation by Chel A in HCT116 cells, whereas the activation of Chk2 and ATR at a relatively early stage revealed their potential participation in p53 protein induction by Chel A treatment. In light of the abrogation of p53 stabilization, as well as the non-disruptive ATR activation in Chk2 knockout cells, Chk2 is very likely to function as a mediator being responsible for p53 accumulation. Moreover, based on well-documented previous studies [10], phosphorylation of p53 at Ser20 may be also induced by Chk2 in our experimental system. Considering that ATR conditional knockout crippled the induction of both phosphorylated p53 and Chk2, we conclude that ATR is not only substantiated to be a key factor for in Chel A-induced p53 protein expression, but also suggest that ATR acts as a Chk2 upstream regulator. This result, together with previous literature [10, 19, 20], suggested that ATR is possible to make binary decisions to mediate p53 protein accumulation following Chel A treatment——that is, either phosphorylating p53 at Ser15 directly or activating Chk2 via the phosphorylation at Thr68, both of which would next lead to p53 protein accumulation. Taken all evidence together, our results indicate that Chel A-induced p53 protein expression in HCT116 cells could be via the bifurcating signaling cascades, i.e. ATR/p53 signaling and ATR/Chk2/p53 axis.

ROS as products of cellular metabolism could potentially result in DNA damage and likewise play an important role in apoptosis induction [18, 21-23]. A host of stimulations have been identified hitherto to augment the intracellular ROS level, thereby triggering apoptosis [24-26]. Herein, we found that Chel A treatment could lead to hydrogen peroxide generation, which was central to Chel A-induced activation of ATR, Chk2, and induction of p53 protein, as induction of ROS generation upon Chel A treatment and the findings that ATR/Chk2 activation and p53 protein expression by Chel A were almost completely impaired by ectopic expression of mCat in HCT116 cells. Furthermore, inhibition of cancer cell anchorage-independent growth was also reversed by mCat overexpression. Collectively, hydrogen peroxide generation in response to Chel A treatment was suggested to be responsible for occuring a series of aforementioned signaling events, cell apoptosis and inhibition of cancer cell anchorage-independent growth. Nevertheless, the detailed molecular mechanism responsible for hydrogen peroxide generation by Chel A remains mysterious, and hence is currently underway in our laboratory.

In summary, our studies demonstrate a chemotherapeutic function of Chel A by triggering apoptosis in HCT116 cells. Further studies on apoptosis induction implicated that hydrogen peroxide generation following Chel A treatment mediates ATR phosphorylation and activation. The activated ATR possessed its p53 protein accumulation through both ATR/p53 cascade or ATR/Chk2/p53 axis, which in turn exclusively lead to apoptosis (Fig. 6G). Thus, the natural compound Chel A would be exploited as a potential chemotherapeutic agent for cancer therapy.

## MATERIAL AND METHODS

### Chemicals

Chel A [6(7,8-epoxy-styryl)-5-acetoxy-5,6-dihydro-2-pyrone] was isolated from *Goniothalamus cheliensis* by the Kunming Institute of Botany, Chinese Academy of Sciences, Kunming, China ref. [5]. Dichlorofluorescein diacetate (DCFH-DA) and hydroethidine (HE) were from purchased from Invitrogen (Carlsbad, CA, USA). Chemicals MG132 and cycloheximide (CHX) were purchased from Calbiochem (San Diego, CA, USA). Dichlorofluorescein diacetate (DCFH-DA) and hydroethidine (HE) were from purchased from Invitrogen (Carlsbad, CA, USA).

### Cell culture

The human colon cancer cell line HCT116 and its p53-deficient (HCT116 p53−/−) derivative were kindly provided by Dr. Bert Vogelstein (The Sidney Kimmel Comprehensive Cancer Center, The Johns Hopkins University Medical Institutions, Baltimore) and used in our previous studies [27-30]. HCT116 Chk2−/− and HCT116 ATR flox/- cells cells were kindly provided by Dr. Jian Wang, West Virginia University [22][32]. The HCT116 cells and their stable transfectant lines were maintained at 37°C in a 5% CO_2_ incubator in McCoy's 5A medium supplemented with 10% fetal bovine serum (FBS), 2 mM L-glutamine, and 25 ¼g/ml gentamicin. The cultures were dissociated with trypsin and transferred to new 25 cm^2^ culture flasks twice a week. FBS was purchased from Life Technologies, Inc. (Gaithersburg, MD, USA), and the other cell culture reagents were obtained from Sigma (St. Louis, MO, USA).

### Plasmids and transfection

Mitochondria Catalase expression plasmid (pZeo/mCat) and its parental control vector were kindly provided by Dr. J. Andres Melendez (Center for Immunology and Microbial Disease, Albany Medical College) as described before [31, 32]. HCT116 cells were transfected with mCat and its corresponding control vector by using PolyJetTM DNA In Vitro Transfection Reagent (SignaGen Laboratories, Rockville, MD, USA) following the manufacturer's instructions and stable transfectants were selected by Zeocin-resistant selection.

### Cell proliferation analysis

HCT116 cells were trypsinized and 2×10^3^ viable cells suspended in 100 ¼l medium containing 10% FBS were seeded into each well of 96-well plates to 80% confluence. After exposure to Chel A, cells were lysed with 50 ¼l lysis buffer. The proliferation of the cells was measured using Cell Titer-Glo® Luminescent Cell Viability Assay kit (Promega, Madison, WI) with a luminometer (Wallac 1420 Victor 2 multilabel counter system). The results were expressed as luminescence activity relative to medium alone (proliferation index)[30, 33].

### Anchorage-independent cancer cell growth

Soft agar colony formation assay was performed as previously described [30, 34, 35]. In brief, 2.5 ml of 0.5% agar in basal modified Eagle's medium (BMEM) supplemented with 10% FBS, and 20 ng/ml EGF, as well as Chel A, at indicated concentrations, was layered onto each well of 6-well tissue culture plates. 3×10^4^ HCT116 cells, their knockout derivatives, or their stable transfectants were mixed with 1 ml of 0.5% agar BMEM supplemented with 10% FBS, as well as with or without Chel A, and layered on top of the 0.5% agar layer. The plates were incubated at 37°C in 5% CO_2_ for three weeks. The colonies were then counted under microscopy. Those with more than 32 cells were scored. The results were presented as colonies/10^4^ seeded cells.

### Western blottings

HCT116 cells and their knockout derivatives or transfectants were cultured in each well of 6-well plates with normal medium until they reached 70%-80% confluence. Cell culture medium was replaced to medium with 0.1% FBS for 24 hours. Then the cells were treated with Chel A with indicated concentrations and time periods. following by being washed with ice-cold PBS, and then extracted with cell lysis buffer (10 mM Tris-HCl, pH 7.4, 1% SDS, 1mM Na_3_VO_4_, and proteasome inhibitor). The cell extracts were subjected to Western blotting with each of the antibodies for determination of PARP, Cleaved PARP, Caspase 3, Cleaved Caspase 3, p53, p-p53 Ser20, p-p53 Ser15, p-MDM2 Ser166, Chk2, p-Chk2 Thr68, p-Chk1 Ser345, ATR, p-ATR Ser428(Cell Signaling, Beverly, MA, USA), Catalase (Calbiochem, EMD Biosciences, Inc., La Jolla, CA, USA), MDM2, β-Actin (Sigma, St. Louis, MO, USA), as indicated. The protein bands specifically bound to the primary antibodies were detected using an alkaline phosphatase-linked secondary antibody and an ECF (enhanced chemifluorescence) Western blotting analysis system (Amersham Pharmacia Biotech, Piscataway, NJ, USA), as previously described [34, 36, 37].

### Reverse transcription polymerase chain reaction (RT-PCR)

HCT116 cells were cultured in 6-well plates until reaching 70%-80% confluence. Cell culture medium was changed to 0.1% FBS medium for 24 hrs. Then cells were exposed to Chel A using the same method as was used for the cells treated for Western blotting assay. After treatment for the indicated time periods, total RNAs were extracted from cells using Trizol reagent (Invitrogen, Carlsbad, California, USA). Total cDNAs were synthesized by using oligdT_(20)_ primer by SuperScript™ First-Strand Synthesis system (Invitrogen, Carlsbad, California, USA). ATR, p53 and β-actin mRNA presented in the cells were determined by semiquantative RT-PCR assay. Human ATR (Forward: 5'-gcgtgcctgccagaatggga-3', Reverse 5'-cgatcg gagcggccagcttt−3'), p53 (Forward: 5'-gaacccttgcttgcaatagg-3', Reverse 5'-gtgaggtaggtgcaaatgcc-3') and β-actin (Forward: 5'-gcgagaagatgacccagatcat-3', Reverse: 5'-gctcaggaggagcaatgatctt-3') primers (Invitrogen) were used to determine the mRNA levels of ATR, p53 and β-actin, respectively [36]. The PCR products were separated onto 3% agarose gels, stained with EB, and scanned the images from a UV light as described previously [36, 38, 39].

### Flow cytometry assay

HCT116 cells were cultured in 6-well plates until 70%–80% confluent. Cell culture medium was replaced with 0.1% FBS medium for 24 hrs. The cells were then treated with Chel A at indicated concentrations. After 48 hrs treatment, cells were washed, and fixed in ice-cold 70% ethanol and stained with PI buffer (0.1% Triton X-100, 0.2 mg/ml RNase A, and 0.05 mg/ml PI) for 15 min. The samples were subjected to flow cytometry (Beckman) for apoptosis analysis as described in our previously[36, 40].

### ROS detection by fluorescence spectrophotometer analysis

HCT116 cells were seeded into 96-well plates until 75-80% confluent. The cell culture medium was replaced with 0.1% FBS McCoy's 5A medium for 24 hrs. The cells were incubated with DCFH-DA or HE for 0.5 hr, and were then exposed to Chel A at indicated time periods followed by washing with PBS to remove the extracellular compounds and the fluorescence Intensities were detected by using SpectroMax M2 Microplate Reader (Molecular Devices Corp.). The cells incubated with DCFH-DA were employed to monitor the production of hydrogen peroxide, with the excitation of 488 nm and emission of 530 nm, while cells treated with HE were used to the measure of superoxide, with excitation of 510 nm and emission of 600 nm [32, 41, 42].

### Statistical analysis

The student's t-test was used to determine the significance between Chel A-treated vs. untreated group, or between specific gene knockout group vs. wild type group, or between specific gene transfectant vs. vector transfectant. The results are expressed as mean ± SD from at least three independent experiments. *P*<0.05 was considered as a significant difference between comparison groups.
